# Hyperspectral Imaging for Bloodstain Identification

**DOI:** 10.3390/s21093045

**Published:** 2021-04-27

**Authors:** Maheen Zulfiqar, Muhammad Ahmad, Ahmed Sohaib, Manuel Mazzara, Salvatore Distefano

**Affiliations:** 1 Department of Computer Engineering, Khwaja Fareed University of Engineering and Information Technology, Rahim Yar Khan 64200, Pakistan; mahiryk2009@gmail.com (M.Z.); ahmed.sohaib@kfueit.edu.pk (A.S.); 2 Department of Computer Science, National University of Computer and Emerging Sciences, Islamabad, Chiniot-Faisalabad Campus, Chiniot 35400, Pakistan; 3 Dipartimento di Matematica e Informatica-MIFT, University of Messina, 98121 Messina, Italy; sdistefano@unime.it; 4 Institute of Software Development and Engineering, Innopolis University, 420500 Innopolis, Russia; m.mazzara@innopolis.ru

**Keywords:** hyperspectral imaging, bloodstains identification, weak bands, SVM, ANNs

## Abstract

Blood is key evidence to reconstruct crime scenes in forensic sciences. Blood identification can help to confirm a suspect, and for that reason, several chemical methods are used to reconstruct the crime scene however, these methods can affect subsequent DNA analysis. Therefore, this study presents a non-destructive method for bloodstain identification using Hyperspectral Imaging (HSI, 397–1000 nm range). The proposed method is based on the visualization of heme-components bands in the 500–700 nm spectral range. For experimental and validation purposes, a total of 225 blood (different donors) and non-blood (protein-based ketchup, rust acrylic paint, red acrylic paint, brown acrylic paint, red nail polish, rust nail polish, fake blood, and red ink) samples (HSI cubes, each cube is of size 1000 × 512 × 224, in which 1000 × 512 are the spatial dimensions and 224 spectral bands) were deposited on three substrates (white cotton fabric, white tile, and PVC wall sheet). The samples are imaged for up to three days to include aging. Savitzky Golay filtering has been used to highlight the subtle bands of all samples, particularly the aged ones. Based on the derivative spectrum, important spectral bands were selected to train five different classifiers (SVM, ANN, KNN, Random Forest, and Decision Tree). The comparative analysis reveals that the proposed method outperformed several state-of-the-art methods.

## 1. Introduction

The handling of a crime scene is an important part of successful and dynamic criminal investigations. Forensic science deals with true crime casework for the collection, detection, and analysis of evidence material. Different traces that are found in crime casework can be very important. A schematic search, analysis, and conclusions from these traces make them valuable in court investigations [1,2,3,4]. One of the most important forms of forensic shreds of evidence found at a crime scene is body fluids. Blood is a valuable and common body fluid found at violent crime scenes [5]. The analysis of bloodstain patterns and the age determination of bloodstains are interesting areas of forensic casework that can lead to identifying suspects [6,7].

In the case of stain detection at a crime scene, the first challenge is to develop a technique for the confirmation of a stain as a bloodstain. This is because a bloodstain can be comparable to other substances in terms of color and appearance on different substrates on visual inspection [8]. Though Deoxyribonucleic Acid (DNA) can be used to identify a suspect, however, DNA analysis is time-consuming and expensive. The presence of false positives like a stain of brown paint may particularly lead to a waste of resources and time. Therefore, true blood stains should be selected for subsequent DNA analysis.

Several presumptive tests are used for the identification of stains as bloodstains against other confusing substances. These tests include chemical methods, for instance, Kastle–Meyer (KM), Leucomalachite Green (LMG), Benzidine [9], and Luminol [10]. The KM test is also known as the phenolphthalein test and it may produce false positives [11]. Although it is not sensitive, it can detect blood after a dilution ratio of 1 in 10,000. LMG is also sensitive as KM with a 1 in 10,000 dilution ratio [12]. The luminol test is very sensitive as compared to the KM test and produces fewer false positives. Moreover, luminol test requires a dark environment [13,14].

As all these tests are presumptive, therefore, confirmatory tests are required to reduce false positives. These confirmatory tests include spectroscopic, chromatographic, microscopic, and crystal tests. All these aforementioned methods may use chemicals or require sample preparation that can be problematic for subsequent analysis like pattern and DNA analysis. Moreover, a small amount of biological traces are available in some cases. Therefore, these tests can destroy the original context [15,16].

Therefore, the forensic work is captivated by non-destructive methods for the identification of shreds of evidence for the recent period [17]. Different non-contact techniques have been used in the forensic area. Practitioners are using different spectroscopic techniques like Raman, Reflectance, Electron Paramagnetic Resonance (EPR), Nuclear Magnetic Resonance (NMR), and Infrared (IR) spectroscopies like Attenuated Total Reflectance Fourier Transform IR spectroscopy (ATR-FTIR). IR and Raman-based techniques provide promising results for bloodstain identification [18].

For instance, Bremmer et al. used reflectance spectroscopy in the visible region to identify blood based on the correlation coefficient [19]. Edelman et al. used spectroscopy in NIR and found difficulty in blood identification in the visible region for colored substrates due to high absorbance. The authors did not include any non-blood substance containing protein to avoid false positives. Moreover, the method was best suitable for dried samples due to the water peak in the NIR [20]. Pereira et al. identified human blood through the evaluation of different supervised pattern recognition techniques with NIR spectroscopy [21]. Morillas et al. used a portable NIR spectrometer for bloodstain identification. The authors observed good results with 81–94% accuracy with different classification models [22].

Likewise, Raman spectroscopy was used to distinguish human and non-human species, for gender identification and to discriminate between infant and adult blood donors. Moreover, ATR-FTIR spectroscopy was used in different forensic applications [18,23,24], but IR technologies can be used only for IR active substances. Though Raman technology has more promising results than IR, in forensic applications, however, its accuracy of forensic work degrades for dried-up samples due to the degradation of blood structures with powered laser lights. Moreover, complications are found in the case of overlaying weak bands while dealing with Raman signals. This may require expertise and significant analysis [25]. All these spectroscopy techniques are limited to provide the spectral information of the whole specimen instead of spatial information of the whole area under observation. Spatial information helps to extract information of samples with different shapes on different backgrounds. For this purpose, spectral imaging has been used widely, which combines the benefits of both imaging and spectroscopy.

Irrespective to Raman spectroscopy, Hyperspectral Imaging (HSI) with different substrates especially dark and patterned has been proposed in [26]. The authors of [26] found that the bloodstains were visible only at few wavelengths in the visible and NIR range. Edelman et al. demonstrated the visualization of bloodstains on black backgrounds using chemo-metrical techniques with visible HSI. The authors also concluded that the bloodstain identification task with other substances was tedious for dark substrates in this range [27]. B. Li et al. proposed a novel approach for bloodstain identification using Soret peak at 415 nm in visible HSI [28]. Cadd et al. used the same criteria of correlation coefficient with the soret band for the identification of blooded fingerprints on white tiles [29] and different colored tiles [30] in their future studies. Cadd et al. extended the work with a chemical enhancement of Acid Black 1 for the first time to detect blood-stained fingerprint [31]. In all of the above, temperature and humidity must be maintained to avoid blue spectral shift [32]. Moreover, the authors reported high accuracy with a soret band instead of weak α and β absorption bands.

Irrespective of the works discussed above, in this work, HSI technology has been used for non-contact identification of blood traces, thus limiting the problem of destruction and contamination of these traces. HSI gives both spatial and spectral information of material under observation [33]. Hence, fast data acquisition, less expected human error, and no sample preparation lessens the load of work in labs and helps in further analysis after quick identification. In the modern era, HSI finds its applications in food quality assessment, medical imaging, security and defense, remote sensing [34,35] field, and artwork authentication [36].

In a nutshell, in this work, a bloodstain is identified against eight different blood resembling substances using the HSI system in the 397–1000 nm range. The blood samples from three donors were imaged, using three different substrates (white cotton fabric, white tile, and PVC wall sheet) with eight different non-blood items (ketchup, rust acrylic paint, red acrylic paint, brown acrylic paint, red nail polish, rust nail polish, fake blood, and red ink). Savitzky Golay derivative has been used to enhance the features of the blood spectrum. The important wavelengths (bands) were used to train and test five different classification models. Finally, a blind experiment has been performed with a blood sample of the fourth blood donor and different blood resembling substances on each substrate for further validation of the proposed methodology.

## 2. Materials and Methods

This section summarizes the sample preparation, hardware system, data acquisition, data pre-processing, and identification criteria with different classification models.

### 2.1. HSI System

HSI system used in this study includes an FX-10 (Specim, Spectral Imaging Ltd, Finland) Hyperspectral camera, equipped with a lens from Scheiner and a line scanner. The setup contained three halogen lamps, a moving platform for scanning, and a camera mounting plate with an adjustable height. A serial communication port was used to connect the scanner directly to a laptop where data was dumped by a software Lumo Scanner. GigE-Vision was interfaced with a camera and laptop to transfer captured data. Three types of raw files including dark reference, white reference, and sample were obtained. For dark and white references, 100 frames were acquired by closing the shutter and using a white tile, respectively. The HSI system is capable of capturing a hypercube of a size of 1000 × 512 × 224 in the visible NIR range between 397–1003 nm with an average subsampling of 2.7 nm.

### 2.2. Sample Preparation

In this study, human blood is used for identification purposes against different blood resembling substances including ketchup, rust acrylic paint, red acrylic paint, brown acrylic paint, red nail polish, rust nail polish, fake blood, and red ink. The blood was stained on different substrates directly from the fingertips after the informed consent of volunteers during the whole experimentation. For this purpose, the ACCU-CHECK® safe lancing device was used with sterilized needles. The needle was disposed of after each blood sample deposition. Before blood sample deposition, all substrates were cleaned with distilled water. The stain size of each blood and non-blood sample were tried to be kept within a diameter of about 1 cm but the number of pixels within one stain could be varied. To apply the same number of stains for each surface, a different number of pieces of each substrate were used according to their available surface area.

The HSI cubes (blood and non-blood samples) were captured for up to three days in order to include dehydrated (aging) samples. The samples were placed to dry for about one hour before imaging on the first day. Then the samples were kept at room temperature in an airtight box. The observed averaged temperature and humidity were 39 °C and 40%, respectively. The precise information about the number of samples (HSI cubes) is shown in Table 1. The details related to number of pixel spectra of all stains and their distribution in different sets are shown in Table 2 and Table 3.

The sample deposition information for three substrates (white cotton fabric, white tile, and wall sheet) is given below:

- Three bloodstains were deposited by each of the three donors, creating a total of nine stains on each substrate. These samples were imaged for three days so making a dataset of 27 samples (HSI cubes) for each substrate. These blood samples together with non-blood samples were used for model training and testing;
- For blood resembling substances, two stains of each substance (ketchup, rust acrylic paint, red acrylic paint, brown acrylic paint, red nail polish, rust nail polish, fake blood, and red ink) were deposited on each substrate and imaged for up to three days as similar to the blood samples. A total of 48 samples (HSI cubes) of 8 different blood resembling substances along with 27 bloodstains were used for training and testing of the models for each substrate;
- Blind Trial: For the final evaluation and validation of trained/tested models, a blind experiment was also conducted on entirely unseen blood samples (HSI cubes). These samples were collected as; each substrate was stained with two blood samples of different aging, from another donor along with four non-blood samples (6 HSI cubes). These HSI cubes are of size 410 × 512 × 224 as compared to the previous experiments which were conducted on 100 × 100 × 224.

### 2.3. Spectral Reflectance

The samples (blood, non-blood stains) were placed on the moving platform of the HSI system. A white tile of 99.9% reflectance placed on a moving platform was used as a white reference. The speed of the platform was set to 11.72 mm/s. The frame rate was 50 Hz and the exposure time was 16 ms. The camera was adjusted at a height of 15 cm. These settings were kept constant for the entire data collection. As the size of each substrate was different, therefore, the number of frames of hypercube was set according to the size of different substrates.

The hyperspectral camera records the radiance of the specimen. The recorded radiance suffers from different factors like a spectrum of the illumination source, incident angle with a specimen, atmospheric effects, shadowing, and sensor effects. Therefore, it is necessary to convert radiance into spectral reflectance with the removal of different factors. For this purpose, two calibration targets, white reference and dark reference, with a wide brightness difference, were used to calculate reflectance from encoded sample radiance by using the Empirical Line Method [37]. Path radiance and shadowing effects are also eliminated with Empirical Line Method. The linear equation is used to calculate spectral reflectance from encoded radiance for each spectral band of a hypercube.

(1) $$Reflectance=\frac{R_{specimen}-D_{ref}}{W_{ref}-D_{ref}}$$

where $R_{specimen}$ is encoded radiance of sample while $D_{ref}$ and $W_{ref}$ are captured dark and white reference frames.

### 2.4. Pre-Processing

**Figure 1:**
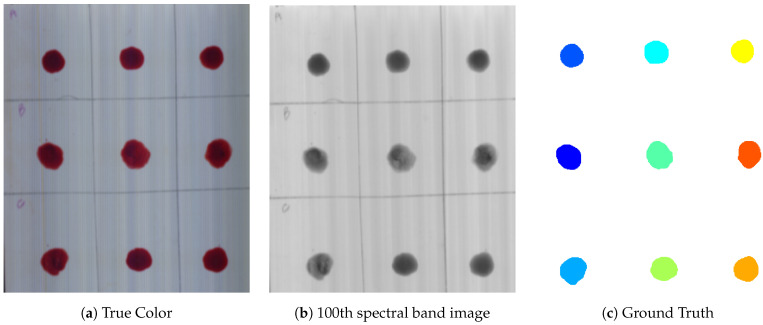
Blood sample deposited on a wall sheet.

The pre-processing was divided into two parts, i.e., spatial and spectral pre-processing. Image pre-processing techniques have been applied in order to extract a Region of Interest (ROI) from an image. For ROI, a Hyperspectral reflectance image of a data cube of 100 × 100 pixels for each blood and non-blood sample was cropped. Then, thresholding was done at 540 nm. After extracting the mask, it was multiplied with the whole hypercube to extract the spectral information of pixels related to ROI as shown in Figure 1. To remove the noise, Standard Normal Variate (SNV), Multiplicative Scatter Correction (MSC), Smoothing with Savitzky Golay method, and averaging filter were used [21,38,39,40].

Based on visual inspection, the Savitzky Golay smoothing filter performed well for noise removal. To deal with pixel information in an HSI cube, a derivative is a helpful technique to detect pixel variations, subtle characteristics, and weak absorption bands. First-order derivatives can be used to remove spectral shifts. However, in this work, higher-order derivatives are used to extract overlapping spectral features and for background elimination [41]. Higher-order derivatives cause a reduction in signal-to-noise ratio (SNR). Savitzky Golay filter is one of the mathematical derivative methods that are better than others due to its soothing property [42,43,44,45]. The Savitzky Golay filter is based on polynomial fitting on data points depending upon window size. The solution of a polynomial is found by the least square minimization. The polynomial order is fixed depending on the derivative order to be calculated [46]. In this study, Savitzky Golay’s second order derivative with a 13-point window and third-order polynomial was used to remove spectral shifts due to aging, irrelevant noise, different donor samples, and pixel variations. The derivative of aged samples also highlighted the subtle dips to make the blood spectrum homogeneous.

### 2.5. Identification Criteria of Blood from other Red Substances

**Figure 2:**
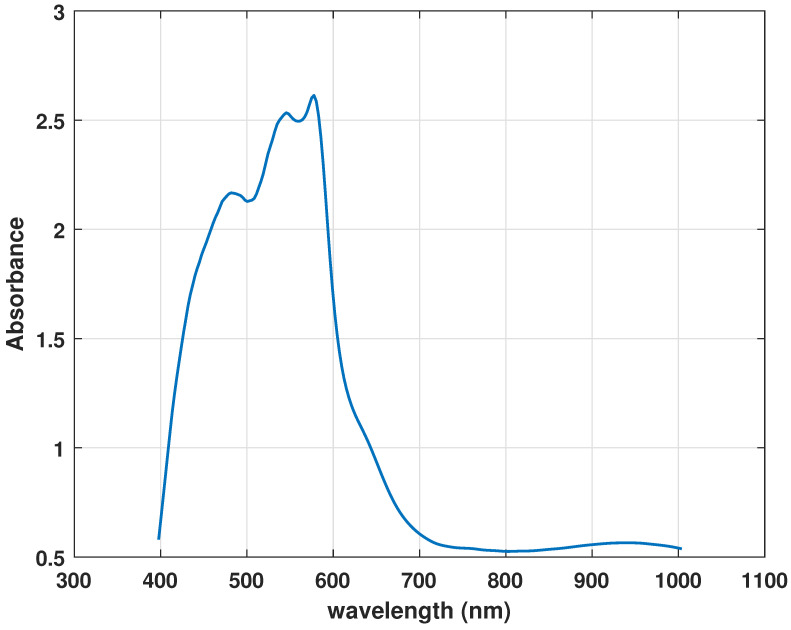
Blood spectrum.

Hemoglobin is an important component of blood. Dried-up bloodstains contain about 97% of hemoglobin components. Oxyhemoglobin, meta-hemoglobin, and hemi-chrome are important hemoglobin derivatives in vitro reactions [47]. The spectral properties of hemoglobin derivatives are used in this study to develop identification criteria. The mean reflectance spectra and their derivatives of blood samples are observed against different red-colored substances that could be confused with blood. In the visible region, two peaks α and β are found due to oxyhemoglobin at wavelengths 577 nm and 540 nm respectively as shown in Figure 2. With aging, the dips become less prominent on visual inspection in a spectral signature.

Moreover, pixel-wise reflectance suffered from spectral noises may cause spectral shifts. The steepness in the curve of blood is observed from 600–650 nm, which is due to the formation of hemi-chrome and meta-hemoglobin, moreover, with aging, this steepness also decreases [48]. As mentioned earlier, the Savitzky Golay derivative is used to make dips and peaks of aged blood samples and non-blood samples more prominent. Moreover, it also highlighted a prominent change in the blood derivative spectrum against a non-blood derivative spectrum between 470–770 nm. In the derivative spectrum, redundant information is ignored, therefore, appropriate spectral bands are selected based on derivative spectra for classification models.

## 3. Spectral Analysis

### 3.1. Pixel Level Spectral Analysis

**Figure 3:**
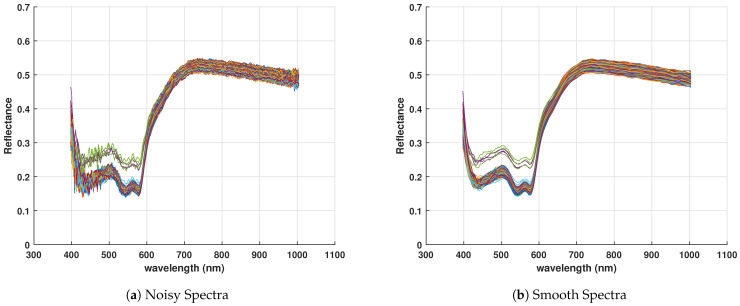
Spectral signature of few pixels: (**a**) Reflectance spectra with noise. (**b**) Reflectance spectra after smoothing.

Illumination source effects, shadowing, atmospheric effects, and uncontrolled stain deposition of samples on substrates may cause variation in pixel values and baseline shifts as shown in Figure 3a. The spectral signature noise has been eliminated by Savitzky Golay smoothing filter as shown in Figure 3b.

### 3.2. Spectral Analysis of Different Blood Donors

**Figure 4:**
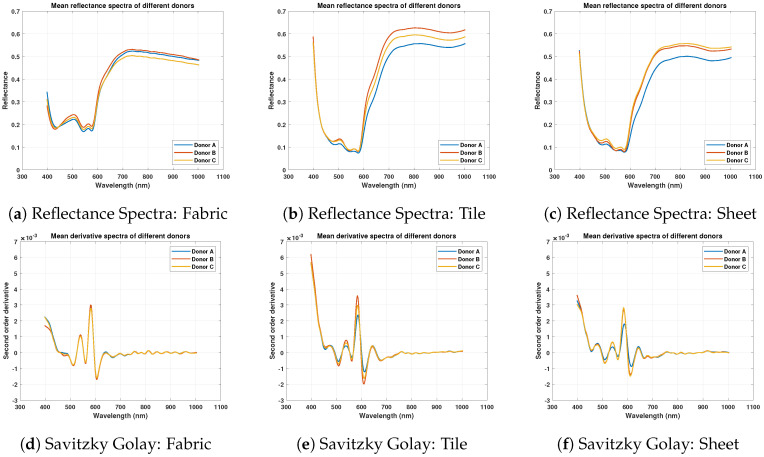
Mean reflectance spectra of different blood donor samples and Savitzky Golay second-order derivative spectra comparison over White Fabric, Whtile Tile, and Wall Sheet.

The mean reflectance spectra of three different donors have been observed that indicate variations in reflectance values. Oxyhemoglobin α and β dips have been observed at 577 nm and 540 nm respectively in the mean reflectance spectra of all samples with little baseline shifts as shown in Figure 4a–c. Moreover, Figure 4a–c show baseline spectral shifts from 700 nm onwards and change in the steepness of slope between 600–650 nm due to different fractions of hemi-chrome and meta-hemoglobin in different samples depending upon environmental conditions.

Moreover, the reflectance spectrum values on cotton fabric are quite different as compared to other substrates. This is due to the absorbing property of the cotton fabric. Savitzky Golay’s second-order derivative has been used to highlight overlapping regions and removes spectral shifts as shown in Figure 4d–f. The derivative spectrum overlaps in most regions and shows peaks and dips in terms of negative and positive values.

### 3.3. Spectral Analysis of Aged Blood Samples

**Figure 5:**
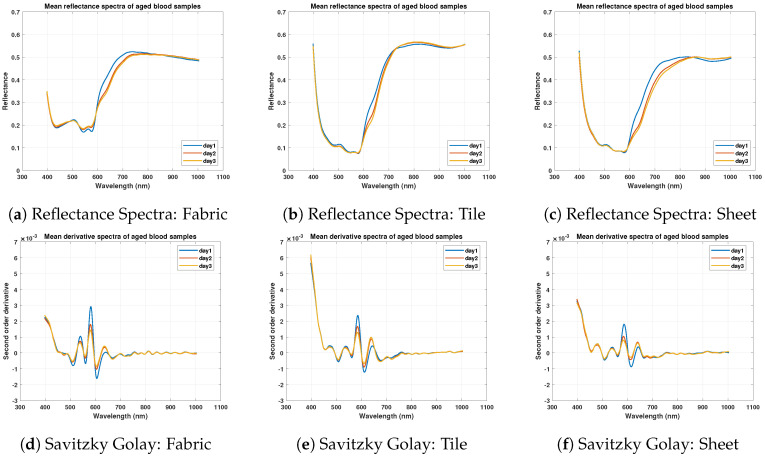
Mean reflectance spectra of aged blood samples of donor 1 and Savitzky Golay second order derivative spectra comparison over white fabric, white tile, and wall sheet.

With the aging of samples, α and β dips in reflectance spectra become smaller and steepness of slope within 600–650 nm decreases as shown in Figure 5a–c. In the case of white tile and wall sheets, the dips have become less prominent. These α and β dips and changes have been observed more clearly with the second-order Savitzky Golay derivative spectrum. Figure 5d–f show that second-order derivative has a homogenized spectrum while enhancing the subtle spectral features with a change in spectrum pattern in a wavelength range of 600–650 nm due to dehydrated samples.

### 3.4. Spectral Analysis of Blood Samples Against Non-Blood Samples

**Figure 6:**
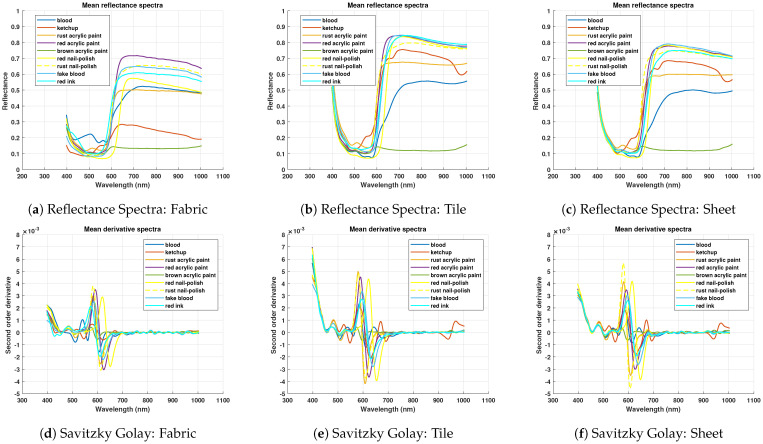
Mean reflectance spectra and Savitzky Golay second order derivative spectra of blood and non-blood samples comparison on white fabric, white tile, and wall sheet.

It has been observed in Figure 6 that the spectral signature of most of the samples overlaps at starting spectral lines. For instance, around 450 nm, small changes can be visualized in the derivative spectrum in Figure 6b,c. From 500 nm onwards, it can be observed that the blood spectrum and rust paint spectrum both have a hump at about 510 nm. Moreover, some non-blood spectra have a light trough around 920–930 nm like blood spectra. All similarities and changes can be visualized clearly in derivative spectra as shown in Figure 6d–f. Each spectral signature shows a steep curve between 580–680 nm, the derivative shows different curvature values for all spectra in this region.

**Figure 7:**
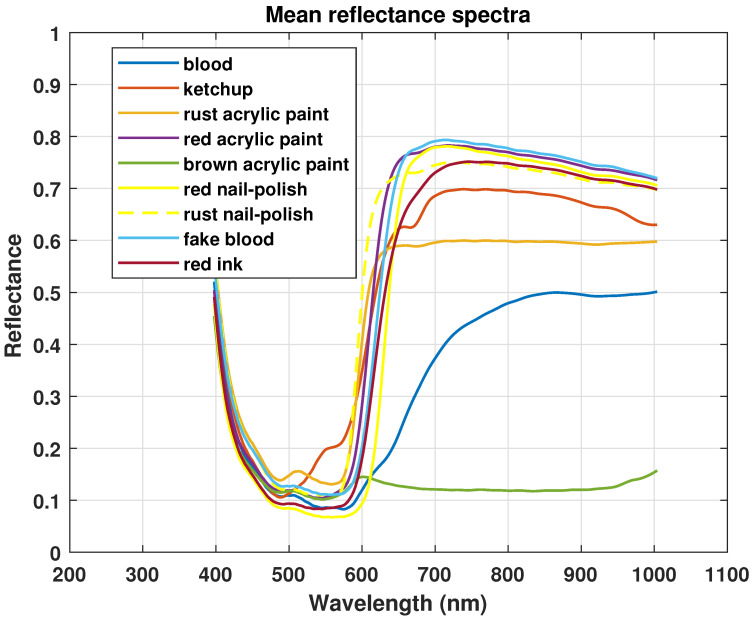
Comparison of spectra of aged blood and different non-blood samples on wall sheet.

In the case of aged samples, as α and β dips become less prominent, therefore blood spectrum becomes flattened like the non-blood spectrum in this region. This can be observed particularly between 500–580 nm for aged samples of day three in Figure 7. The subtle bands of all samples get highlighted in their derivative spectrum. Redundant information has been observed from 800 nm onwards due to the small values of spectra. As blood derivative spectra have a prominent difference between 470–770 nm, therefore, these spectral lines have been selected for model training.

## 4. Data Splitting

In this study, the individual pixels of each stain has been treated as observations. The acquired data has been split into training, validation, and test sets in two following ways while using the holdout cross-validation technique:

- In the first case, 70% of bloodstains have been used as a training data set while 30% for the external validation data set. It has been noticed that the stains of each aging have been included in training and test sets. From training samples, 80% samples have been used for model training and 20% for internal validation. A similar procedure has been done with non-blood samples. The shuffling has been done for sample division. In all these samples, the number of pixels varies due to small variations in ROI size. The breakdown of all data sets has been shown in terms of total observations in Table 2;
- In the second case, 70% of pixels of each blood and non-blood stain have been included in the training data set while 30% of pixels being in the external validation data set. Then 20% of training pixels of each stain, have been used for internal validation. The shuffling of pixels within each stain has been done before concatenation. The division of observations is shown in Table 3.

## 5. Experimental Settings and Results

For experimental evaluation, several statistical tests have been conducted including but not limited to Kappa (κ), overall accuracy, sensitivity, specificity, F1-score, precision, and recall rate. All these evaluation metrics are calculated using the following mathematical formulations.

(2) $$OA=\frac{1}{C}\sum_{i=1}^{C}TP_{i}$$

(3) $$\kappa =\frac{P_{o}-P_{e}}{1-P_{e}}$$

where

(4) $$P_{o}=\frac{TP+TN}{TP+FN+FP+TN}$$

(5) $$P_{e}=P_{Y}+P_{N}$$

(6) $$P_{Y}=\frac{TP+FN}{TP+FN+FP+TN}\times \frac{TP+FN}{TP+FN+FP+TN}$$

(7) $$P_{N}=\frac{FP+TN}{TP+FN+FP+TN}\times \frac{FN+TN}{TP+FN+FP+TN}$$

where *TP* and *FP* are true and false positive, and *TN* and *FN* are true and false negative computed from the confusion matrix.

(8) $$Precision=\frac{1}{C}\sum_{i=1}^{C}\frac{TP_{i}}{TP_{i}+FP_{i}}$$

(9) $$Sensitivity=Recall=\frac{1}{C}\sum_{i=1}^{C}\frac{TP_{i}}{TP_{i}+FN_{i}}$$

(10) $$F1-Score=\frac{2\times (Recall\times Precision)}{\left(Recall+Precision\right)}$$

(11) $$specificity=\frac{1}{C}\sum_{i=1}^{C}\frac{TN_{i}}{TN_{i}+FP_{i}}.$$

Experiments have been conducted using five different types of classifiers, i.e., Support Vector Machine (SVM) [49], K Nearest Neighbors (KNN) [49], Artificial Neural Network (ANN) [50], Decision Trees (DT) [51], and Random Forest (RF) [51]. The tuning parameters for all the aforementioned classifiers are as follows: In the case of ANNs, a dense network with three hidden layers, each containing 30 neurons, was used. The number of layers and nodes were selected by the hit and trial method. In order to increase sensitivity and specificity, the number of epochs was increased up to 50. SVM is trained using Linear and RBF kernel functions and rest of the parameters are left default. KNN was tuned between [1, 20] nearest neighbors iteratively. DT was trained using entropy criterion with 10 times depth and finally, and RF was trained using 500 estimators.

The tuning parameters of all the above-said classifiers were explored very carefully in the first few experiments (as listed above) and those that provided the best accuracy were chosen. To avoid bias, all the listed experiments are carried out in the same settings on the same machine. Before the experiments, we performed the necessary normalization between [0, 1], and all the experiments are carried out using Matlab 2021a installed on an Intel inside Core i3-4030U CPU 1.90 GHz with 12 GB RAM.

As discussed in Section 4, classification was performed with two separate data sets. For feature reduction, Principle Component Analysis (PCA) was performed on each sample data with a selection of all wavelengths which significantly reduces the performance of SVM even with different kernels functions. Then, the results were observed with a second-order derivative spectrum with two spectral ranges i.e., 397–770 nm and 470–770 nm. Similar results have been drawn with both spectral ranges, therefore, a 470–770 nm spectral range was selected to train models with a fewer number of features which improves the generalization performance. It has been observed that all the classifiers performed with 100% accuracy and statistical significance for all blood samples as given in Table 4 and Table 5.

Similarly, blood vs. blood (donor A, B, and C as a separate class) vs. 8 other non-blood samples (protein-based ketchup, rust acrylic paint, red acrylic paint, brown acrylic paint, red nail polish, rust nail polish, fake blood, and red ink) on three different substrates (white cotton fabric, white tile, and PVC wall sheet) as multi-class classification results have also been carried to show the performance of our proposed pipeline. Moreover, the ageing of all these results have also been discussed in Table 6, Table 7 and Table 8, respectively.

## 6. Blind Testing

**Figure 8:**
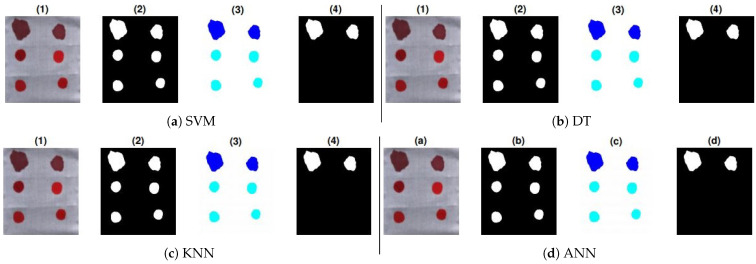
Prediction results of test samples on white fabric: (1) True color image, (2) pre-processed image, (3) labeling, and (4) classifier results: (**a**) SVM, (**b**) DT, (**c**) KNN, and (**d**) ANN.

**Figure 9:**
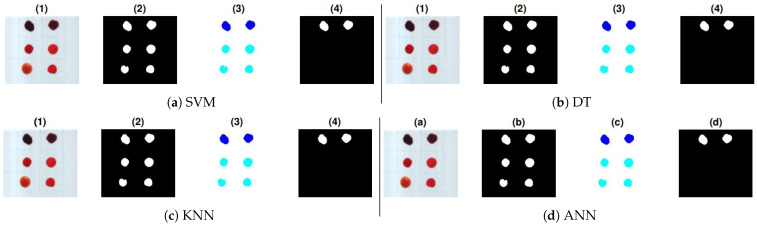
Prediction results of test samples on white tile: (1) True color image, (2) pre-processed image, (3) labeling, and (4) classifier results: (**a**) SVM, (**b**) DT (**c**) KNN, and (**d**) ANN.

**Figure 10:**
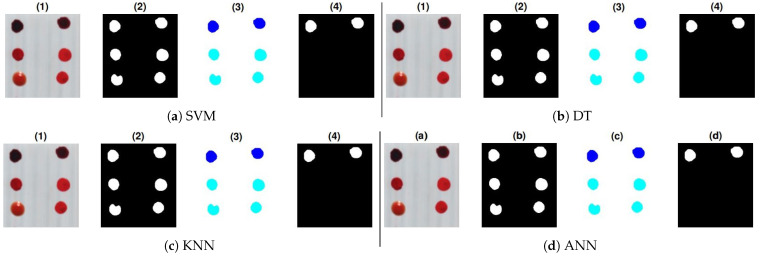
Prediction results of test samples on wall sheet: (1) True color image, (2) pre-processed image, (3) labeling, (4) classifier results: (**a**) SVM, (**b**) DT, (**c**) KNN, and (**d**) ANN.

Further validation of the proposed work is performed with the application of new samples (HSI cubes) on all substrates. To this aim, two bloodstains of the fourth donor with different aging have been deposited in the top row of each substrate and also 4 non-blood samples have been deposited on each substrate as mentioned in Section 4. The location of the blood samples was not mentioned before testing. The ground truths of these samples are shown in Figure 8, Figure 9 and Figure 10. These show prediction results with different classification models. The blood extraction rate is found to be 100% with the purposed methodology.

## 7. Comparison with State-of-the-Art Methodologies

In the literature, PCA is a commonly used method for feature reduction. PCA is used to map high-dimensional data to lower dimensions while taking a linear combination of original data with orthogonal vectors known as Principal Components (PCs) [52]. The top PCs of transformed data carry as much variation as possible. To strengthen our proposed pipeline, feature reduction has been performed with PCA instead of derivatives. We have trained our models with the first three PCs for comparison.

Moreover, study [21] evaluated different supervised pattern recognition methods for blood identification. This study followed Standard Normal Variate (SNV) and Normalization in the pre-processing step. We have followed their two pipelines with Soft Independent Modeling of Class Analogy (SIMCA) and Partial-Least Squares Discriminant Analysis (PLS-DA) models to compare with our results. The number of PCs selected for both models is 3 and 2, respectively. Table 9 presents the results of these methodologies against our proposed pre-processing method and derivative-based feature selection. These results have been drawn with the same data set used in this study.

The results have been presented for the identification of blood against eight different blood resembling substances using HSI technology in the visible region. These blood and non-blood samples were deposited on different substrates that are commonly discussed in forensic applications. These substrate materials included white cotton fabric, white tile, and a PVC wall sheet.

In order to reconstruct old crime scenes, a criterion of aging for three days was set. Therefore, the images were captured for up to three days. The reflectance spectra of all blood and non-blood samples were analyzed visually and the identification criteria are set based on blood heme-components. First, PCA was used for feature reduction from the reflectance spectrum and classification results were observed. To observe reduced performance with PCA, the distribution of transformed data with aged samples is also analyzed. The distribution is randomly scattered due to the change in the chemical composition of different substances. Then Savitzky Golay derivative filter is used in the pre-treatment step to enhance features of aged samples. Therefore, derivative-based important spectral lines are selected while discarding redundant information. The performance of different models used in this study increased to 100% with derivative-based features. The proposed methodology provided the considerable potential to identify blood with Hyperspectral imaging.

## 8. Conclusions

This work presented a bloodstain identification method on different substrates. The proposed method could identify blood samples with aging up to 3 days. The non-blood samples included protein-based ketchup, rust acrylic paint, red acrylic paint, brown acrylic paint, red nail polish, rust nail polish, fake blood, and red ink. The proposed method is based on the enhancement of weak bands in the visible region to discriminate blood from different red-colored substances. Important spectral bands were selected from the derivative spectrum. Machine learning models were used for classification and 100% statistical significance was achieved.

This research work is limited to human blood and could be extended with animal blood samples and more blood resembling substances with a wide range of substrates. To reconstruct old crime scenes, the aging criteria could also be extended. Moreover, several active/self/interactive-learning frameworks could also be tested while considering the limited availability of labeled training samples. Moreover, several 3D [53] and hybrid models [54] could also be included to reduce the efforts of handcrafted features.

## Figures and Tables

**Table 1:** The number of stains on each substrate: In total 81 blood and 144 non-blood stains are collected. In total 225 samples (HSI cubes) are collected for an experimental setup in which each HSI cube is later reduced to 100 × 100 × 224 after extracting the Region of Interest (ROI).

Substrates	Blood Stains			Non-Blood Stains			Total
	Day 1	Day 2	Day 3	Day 1	Day 2	Day 3	
White Fabric	9	9	9	16	16	16	75
White Tile	9	9	9	16	16	16	75
Wall Sheet	9	9	9	16	16	16	75
Total	27	27	27	48	48	48	225

**Table 2:** Samples-based data splitting.

Substrates	Blood Stains		Non-Blood Stains		Total
	Training	Test	Training	Test	
White Fabric	21,841	15,216	48,722	23,393	109,172
White Tile	30,732	12,006	40,057	20,620	103,415
Wall Sheet	24,318	14,827	37,223	16,818	93,186

**Table 3:** Pixel-based data splitting.

Substrates	Blood Stains		Non-Blood Stains		Total
	Training	Test	Training	Test	
White Fabric	25,926	11,131	50,459	21,656	109,172
White Tile	29,905	12,833	42,449	18,228	103,415
Wall Sheet	27,389	11,756	37,807	16,234	93,186

**Table 4:** Blood (donor 1) vs. non-blood experimental results over sample-based data splitting. Similar results have observed for other donors.

Classifier	Spectral Lines	Sensitivity	Specificity	F1-Score	Precision	OA	Kappa (*κ*)
**Substrate: White Fabric**						
SVM linear	110	100%	100%	100%	100%	100%	100%
SVM rbf	110	100%	100%	100%	100%	100%	100%
SVM cubic	110	100%	100%	100%	100%	100%	100%
ANNs	110	100%	100%	100%	100%	100%	100%
DT	110	100%	100%	100%	100%	100%	100%
RF	110	100%	100%	100%	100%	100%	100%
KNN	110	100%	100%	100%	100%	100%	100%
	**Substrate: White Tile**						
SVM linear	110	100%	100%	100%	100%	100%	100%
SVM rbf	110	100%	100%	100%	100%	100%	100%
SVM cubic	110	100%	100%	100%	100%	100%	100%
ANNs	110	100%	100%	100%	100%	100%	100%
DT	110	100%	100%	100%	100%	100%	100%
RF	110	100%	100%	100%	100%	100%	100%
KNN	110	100%	100%	100%	100%	100%	100%
	**Substrate: Wall Sheet**						
SVM linear	110	100%	100%	100%	100%	100%	100%
SVM rbf	110	100%	100%	100%	100%	100%	100%
SVM cubic	110	100%	100%	100%	100%	100%	100%
ANNs	110	100%	100%	100%	100%	100%	100%
DT	110	100%	100%	100%	100%	100%	100%
RF	110	100%	100%	100%	100%	100%	100%
KNN	110	100%	100%	100%	100%	100%	100%

**Table 5:** Blood (donor 1) vs. non-blood experimental results over pixel based data splitting. Similar results are observed for other donors.

Classifier	Spectral Lines	Sensitivity	Specificity	F1-Score	Precision	OA	Kappa (*κ*)
**Substrate: White Fabric**						
SVM linear	110	100%	100%	100%	100%	100%	100%
SVM rbf	110	100%	100%	100%	100%	100%	100%
SVM cubic	110	100%	100%	100%	100%	100%	100%
ANNs	110	100%	100%	100%	100%	100%	100%
DT	110	100%	100%	100%	100%	100%	100%
RF	110	100%	100%	100%	100%	100%	100%
KNN	110	100%	100%	100%	100%	100%	100%
	**Substrate: White Tile**						
SVM linear	110	100%	100%	100%	100%	100%	100%
SVM rbf	110	100%	100%	100%	100%	100%	100%
SVM cubic	110	100%	100%	100%	100%	100%	100%
ANNs	110	100%	100%	100%	100%	100%	100%
DT	110	100%	100%	100%	100%	100%	100%
RF	110	100%	100%	100%	100%	100%	100%
	**Substrate: Wall Sheet**						
2-8 SVM linear	110	100%	100%	100%	100%	100%	100%
SVM rbf	110	100%	100%	100%	100%	100%	100%
SVM cubic	110	100%	100%	100%	100%	100%	100%
ANNs	110	100%	100%	100%	100%	100%	100%
DT	110	100%	100%	100%	100%	100%	100%
RF	110	100%	100%	100%	100%	100%	100%
KNN	110	100%	100%	100%	100%	100%	100%

**Table 6:** Day-1: Blood vs. blood samples experimental results with full wavelengths. The best accuracies are emphasized.

Classifier	Spectral Lines	Train Time	Test Time	Kappa (*κ*)	OA	AA
**Substrate: White Fabric**					
SVM linear	224	26.962	23.110	91.13	92.09	93.37
SVM rbf	224	37.445	33.582	89.50	90.63	91.99
ANNs	224	110.033	0.073	**92.02**	**92.88**	**93.99**
DT	224	27.614	0.016	87.65	88.98	90.45
RF	224	279.00	2.20	87.72	89.04	90.86
KNN	224	4.256	16.17	88.93	90.12	91.71
	**Substrate: White Tile**					
SVM linear	224	46.275	21.99	91.88	92.77	94.67
SVM rbf	224	57.631	42.710	86.72	88.18	91.07
ANNs	224	52.129	0.080	**94.88**	**95.45**	**96.51**
DT	224	24.156	0.037	80.31	82.46	86.84
RF	224	275.992	2.088	89.84	90.96	93.11
KNN	224	3.038	7.559	84.15	85.90	89.28
	**Substrate: Wall Sheet**					
SVM linear	224	46.184	22.780	87.97	89.32	92.89
SVM rbf	224	55.783	39.515	82.76	84.71	88.87
ANNs	224	70.783	0.047	**90.90**	**91.93**	**94.54**
DT	224	24.02	0.008	78.27	80.72	86.20
RF	224	281.383	2.482	84.21	86.00	90.56
KNN	224	2.906	8.991	79.61	81.91	87.56

**Table 7:** Day-2: Blood vs. blood samples experimental results with full wavelengths. The best accuracies are emphasized.

Classifier	Spectral Lines	Train Time	Test Time	Kappa (*κ*)	OA	AA
**Substrate: White Fabric**					
SVM linear	224	31.798	24.953	91.65	92.55	93.55
SVM rbf	224	39.953	42.652	87.86	89.17	90.50
ANNs	224	79.619	0.075	**92.09**	**92.94**	**93.97**
DT	224	26.072	0.016	86.35	87.83	89.19
RF	224	263.760	2.194	87.11	88.49	90.38
KNN	224	3.792	17.20	86.75	88.17	90.04
	**Substrate: White Tile**					
SVM linear	224	34.55	18.076	**94.49**	**95.10**	**96.19**
SVM rbf	224	58.816	44.363	89.42	90.58	92.83
ANNs	224	50.577	0.061	93.88	94.55	95.85
DT	224	26.655	0.016	81.61	83.63	87.40
RF	224	291.066	2.597	91.11	92.09	93.84
KNN	224	3.476	7.411	86.05	87.59	90.54
	**Substrate: Wall Sheet**					
SVM linear	224	47.341	26.604	85.91	87.46	91.16
SVM rbf	224	65.917	48.977	79.24	81.57	85.98
ANNs	224	68.264	0.064	**89.80**	**90.93**	**93.74**
DT	224	22.737	0.024	77.25	79.80	84.83
RF	224	275.39	2.26	82.27	84.25	88.79
KNN	224	2.858	7.308	78.38	80.78	86.27

**Table 8:** Day-3: Blood vs. blood samples experimental results with full wavelengths. The best accuracies are emphasized.

Classifier	Spectral Lines	Train Time	Test Time	Kappa (*κ*)	OA	AA
**Substrate—White Fabric**					
SVM linear	224	28.074	22.686	**91.85**	**92.73**	**93.84**
SVM rbf	224	36.985	39.590	88.18	89.46	91.00
ANNs	224	69.574	0.079	91.15	92.10	93.38
DT	224	24.212	0.014	86.99	88.39	90.10
RF	224	283.086	2.227	87.63	88.96	90.84
KNN	224	3.994	17.963	86.93	88.33	90.32
	**Substrate—White Tile**					
SVM linear	224	39.045	23.161	93.58	94.27	95.44
SVM rbf	224	65.709	47.316	83.43	85.20	88.18
ANNs	224	53.424	0.062	**93.78**	**94.45**	**95.38**
DT	224	24.513	0.010	77.38	79.76	83.92
RF	224	270.871	2.074	89.69	90.79	92.40
KNN	224	2.990	6.073	85.70	87.24	89.65
	**Substrate—Wall Sheet**					
SVM linear	224	50.423	26.396	86.00	87.57	91.53
SVM rbf	224	62.824	40.817	80.00	82.27	87.09
ANNs	224	71.753	0.093	**89.53**	**90.70**	**93.69**
DT	224	24.612	0.011	75.48	78.29	84.06
RF	224	256.58	1.924	82.39	84.38	89.23
KNN	224	2.660	8.359	77.41	79.95	86.06

**Table 9:** Comparison of a proposed pipeline with state-of-the-art methodologies in which SNV stands for Standard Normal Variate, SIMCA stands for Soft Independent Modeling of Class Analogy, and PLS-DA stands for Partial-Least Squares Discriminant Analysis.

Methodology	Accuracy	Sensitivity	Specificity	F1-Score	Precision	Kappa (*κ*)
**Substrate—White Fabric**						
SNV + Normalization + SIMCA	99.5%	98.9%	99.9%	99.9%	99.4%	99.1%
SNV + Normalization + PLS-DA	97.1%	100%	95.2%	93.1%	96.4%	94%
Smoothing + PCA + SVM	83.3%	66.1%	94.5%	88.8%	75.7%	63.5%
Smoothing + Derivative-based feature selection + SVM	100%	100%	100%	100%	100%	100%
Smoothing + Derivative-based feature selection + ANN	100%	100%	100%	100%	100%	100%
Smoothing + Derivative-based feature selection + KNN	100%	100%	100%	100%	100%	100%
Smoothing + Derivative-based feature selection + DT	100%	100%	100%	100%	100%	100%
Smoothing + Derivative-based feature selection + RF	100%	100%	100%	100%	100%	100%
**Substrate—White Tile**						
SNV + Normalization + SIMCA	99.7%	99.9%	99.7%	99.4%	99.6%	99.4%
SNV + Normalization + PLS-DA	95.3%	99.7%	92.8%	88.9%	93.9%	90.2%
Smoothing + PCA + SVM	87.4%	100%	80%	74.4%	85.3%	74.6%
Smoothing + Derivative-based feature selection + SVM	100%	100%	100%	100%	100%	100%
Smoothing + Derivative-based feature selection + ANN	100%	100%	100%	100%	100%	100%
Smoothing + Derivative-based feature selection + KNN	100%	100%	100%	100%	100%	100%
Smoothing + Derivative-based feature selection + DT	100%	100%	100%	100%	100%	100%
Smoothing + Derivative-based feature selection + RF	100%	100%	100%	100%	100%	100%
**Substrate—Wall Sheet**						
SNV + Normalization + SIMCA	99.5%	99.9%	99.1%	99%	99.4%	99%
SNV + Normalization + PLS-DA	95.2%	92.8%	97.2%	96.6%	94.7%	90.2%
Smoothing + PCA + SVM	92.8%	97.3%	88.8%	88.4%	92.6%	85.6%
Smoothing + Derivative-based feature selection + SVM	100%	100%	100%	100%	100%	100%
Smoothing + Derivative-based feature selection + ANN	100%	100%	100%	100%	100%	100%
Smoothing + Derivative-based feature selection + KNN	100%	100%	100%	100%	100%	100%
Smoothing + Derivative-based feature selection + DT	100%	100%	100%	100%	100%	100%
Smoothing + Derivative-based feature selection + RF	100%	100%	100%	100%	100%	100%

## Data Availability

The data presented in this study are available on request from the corresponding author.
